# Supplementary material for: Patterns of Intron Gain and Loss in Fungi
Source: PLoS Biol. 2004 Nov 30;2(12):e422. doi: 10.1371/journal.pbio.0020422 (PMC532390; doi:10.1371/journal.pbio.0020422)
Supplement: Table S1 — Also available at http://genes.mit.edu/NielsenEtAl/. (4.3 MB ZIP). [file pbio.0020422.st001.zip › NielsenEtAl/html/879.html]

AN4625.1.NCU05616.1.MG08029.1.FG04845.1


```
 CLUSTAL W (1.82) Multiple Sequence Alignments - Introns Inserted


Sequence 1: AN4625.1	282 aa
Sequence 2: FG04845.1	299 aa
Sequence 3: NCU05616.1	338 aa
Sequence 4: MG08029.1	313 aa
Alignment Length: 357 aa
Number Identitical Residues: 97 aa
Alignment Score (without introns) 4925


MG08029.1 	---MDPQKIYDQVRAHYSAASQGTTA-------AYGSAIAKSFGYSAEELAHVPEGSNLG
NCU05616.1	MDTRNTSQIYDEVAKHYSAASQTTSV-------KYGETVAKSFGYTEEELANIPEGANLG
FG04845.1 	---MDSKEIYEQVHKRYGSVTKSTTG-------KYEQTVAKAFGYTEEELAGIPEDANLG
AN4625.1  	----MIQDTYSEVQSRYGSIAKQTNADTNENKDDTEDKLAQAFGYTAAELSSLPGKANLG
          	      .. *.:*  :*.: :: *. .:.....   . :*::***:  **: :*  :***

MG08029.1 	LSCGNPLAIASVGE0GETVIDLGSGAGFDAFAAAEKAGPSGNVIGVDMNE0DMLARAS--
NCU05616.1	LSCGNPLAITSLRE0GETVIDLGSGAGFDVFLAATRVGPTGRSIGIDFND~DMLARANSL
FG04845.1 	LSCGNPTALAKLRE0GETVIDLGSGAGFDVLTAAKKVGPAGKAIGVDMNK0NMIDKA--N
AN4625.1  	LSCGNPIAFANLKP0GETIVDLGSGGGIDVFLAARKVGPEGRAIGVDMTE0EMITLATTN
          	****** *::.:   ***::*****.*:*.: ** :.** *. **:*:.. :*:  *.: 

MG08029.1 	EIKAKLDKTNVEFVKSSITDISILESGIADCIISNCVINLVPAAEKHLVFKEIFRLLKPG
NCU05616.1	KSSRGFPDSQVSFLKGSITAIPLESG-TADCIISNCVINLVPEAEKPLVFKEMHRVLKSG
FG04845.1 	ANKAAMKISNAGFIQGVITSVPLSDD-AADCIISNCVINLVPASEKQLVFNEMFRILTPG
AN4625.1  	AKKANFPNNQVQFIKAPITSIPLPDS-SADCIISNCVINLVPKDAKPIVFAEIARLLKPG
          	  .  :  .:. *::. ** :.: ..  **************   * :** *: *:*..*

MG08029.1 	GRLAVSDILAKKPMPEKIRSDIALYVGCISGASTVSEYEEFLKDAGFK---E1ILISDSK
NCU05616.1	GRVAVSDILAKKPLPEKLRNDIAMYVGCIAGASEKQQYETWLKEAGFE---Q~VLIQDTG
FG04845.1 	GRVAISDILARKEFTDEIRNNIALYVGCIAGASPVGDYETFLKNAGFQGELN1ILIVDTK
AN4625.1  	GRVAISDILARKPLSPAFVSDIALYVGCVAGASLVEEYEDWLGRAGLK---D1VLIVDTK
          	**:*:*****:* :.  : .:**:****::***   :** :*  **::   : :** *: 

MG08029.1 	GDLNVYTQIGEDGLKKTDCCASTKAKNEESDGRCKPSSDTNVGLVETDLNQWAE1TKIYN
NCU05616.1	ADINVYLDTDENGNRGGCCAPPGTVKEEEEEKSSSCCAPKKTECAPAGAAGAAA~AAPSC
FG04845.1 	SDLNVYFTAQENES----SCCGNEQTNPACGAGLSTCAPQNSSTELADGQDILG~NRLG-
AN4625.1  	ADINLYKQTLD--------------SSPAD----SCCAPKES-------VQIPA~ANFS-
          	.*:*:*    :              ..       . .:  :                   

MG08029.1 	-----------------------------TEDSPMVTHLNISSA~LTILYESKIAGFGIP
NCU05616.1	CKPAAETTPPCCKPVEKKESSCCKPAAKPEVEAEEKEDLNEWVG~SYKIYAVKN------
FG04845.1 	-----------------------------------IADFNEWAG1SFQVYAVKPMSI---
AN4625.1  	-----------------------------------DVDFNEWVG1SFQIYAVKGGF----
          	                                     .:*   .    :*  *       

MG08029.1 	GI
NCU05616.1	--
FG04845.1 	--
AN4625.1  	--
          	
```
